# Synthesis of Novel Non-Isocyanate Polyurethane/Functionalized Boron Nitride Composites

**DOI:** 10.3390/polym14193934

**Published:** 2022-09-20

**Authors:** Said El Khezraji, Manal Chaib, Suman Thakur, Mustapha Raihane, Miguel A. Lopez-Manchado, Raquel Verdejo, Mohammed Lahcini

**Affiliations:** 1 IMED-Lab, Faculty of Sciences and Techniques, Cadi Ayyad University, Avenue Abdelkrim Elkhattabi, B.P. 549, Marrakech 40000, Morocco; 2 Instituto de Ciencia y Tecnologia de Polimeros (ICTP), CSIC, C/Juan de la Cierva, 3, 28006 Madrid, Spain; 3 Chemical & Biochemical Sciences (CBS), Mohammed VI Polytechnic University, Lot 660, Hay Moulay Rachid, Ben Guerir 43150, Morocco

**Keywords:** polyhydroxyurethanes, boron nitride, non-isocyanate polyurethane, polymer composite, functionalization

## Abstract

Poly(hydroxyurethanes) (PHUs) have been suggested as isocyanate-free, low-toxicity alternatives to polyurethanes (PUs). However, PHUs present low mechanical properties due to the presence of side reactions that limit the production of high-molar mass polymers. Here, we present the synthesis under mild conditions and atmospheric pressure of bi-cyclic carbonate monomer for the production of PHU nanocomposites with good physical properties. The kinetics of the bi-cyclic carbonate synthesis and its complete conversion to urethane were followed by FTIR. The addition of functionalized boron nitrate (f-BN) with sucrose crystals improved the thermal degradation temperature as well as the glass transition by approximately 20 °C and 10 °C, respectively. The storage modulus of PHU films gradually increases with the concentration of f-BN in the composite.

## 1. Introduction

The increasing regulations in toxic or hazardous chemicals are driving the search for new synthetic routes to everyday materials. Among them, polyurethanes (PUs) are of particular interest for their uses as elastomers, adhesives, coatings, and foams [1]. Commonly, PUs are prepared by a polyaddition reaction of polyols and isocyanates, whereas isocyanates are highly toxic in nature, causing global warming and health risks as they are obtained from amine and phosgene [2]. Therefore, research on non-isocyanate polyurethanes (NIPUs) is increasing as one of the most efficient, environmentally friendly, and applicable approaches [3].

One of the most attractive NIPU routes is the cyclic carbonate aminolysis resulting in poly(hydroxyurethane) (PHU) derivatives [2,4,5,6]. PHUs are mostly synthesized from the reaction of a bis-amine with a bi-cyclic carbonate, which in turn is produced from the cyclocarbonation of a bis-epoxide with carbon dioxide (CO_2_) [7,8,9]. A drawback of PHUs is the high density of hydrogen bonds and the presence of side reactions, which limits the production of high-molar mass PHUs with good mechanical properties. Thus, hybrid PHUs have been proposed to overcome these problems, ranging from copolymerization or prepolymer strategies to the preparation of composites [10,11].

Studies of PHU nanocomposites are still scarce, but they have already shown improved thermal and mechanical properties, adhesion performances, and shape memory [10]. Although the main used fillers for PHU nanocomposites have been zero dimensional nanoparticles (silica [12,13,14], polyhedral oligomeric silsesquioxanes [15,16], ZnO [17], and Fe_3_O_4_ [18]), both one-dimensional nanomaterials, (particularly carbon nanotubes [19,20] and nanocellulose [21]) and two-dimensional nanomaterials (nanoclay [22,23] and graphene [24,25]), have also been studied. Here, we investigate the incorporation of functionalized hexagonal boron nitride (f-BN) into PHU to provide high mechanical and thermal properties. Indeed, BN nanomaterials have a high Young modulus (0.8 TPa), superior fracture strength (165 GPa), high chemical and thermal stability (up to 800 °C in the air), and outstanding thermal conductivity (300–2000 Wm^−1^ K^−1^) [26,27,28,29]. Unlike graphene, BN nanomaterial is a dielectric material, with a wide bandgap (~5.6 eV), that has been proposed to be used in a wide range of applications in photocatalysis, pollutant degradation, photovoltaics, or sensors among others [26,27]. Reviews on BN and its composites are available in the literature, highlighting the interest of this nanoparticle [26,27,28], though no previous work has been found on PHU/BN systems. Because nanoparticle functionalization provides greater improvements than their non-functionalized counterparts [10], we choose a sugar-assisted mechanochemical exfoliation (SAMCE) process [29]. SAMCE is an efficient, green, and low-cost process that introduces sucrose moieties and hydroxyl and amine (-OH and -NHx) groups. These PHU/f-BN nanocomposites are then characterized by means of thermal and dynamic mechanical analysis.

## 2. Materials and Methods

### 2.1. Materials

EPIKOTE^TM^ MGS^TM^ RIMR135, a bisphenol-A-(epichlorohydrin) resin (number average molecular weight ≤ 700)-1,6-bis(2,3-epoxypropoxy) hexane, was purchased from Hexion. Tetrabutylammonium bromide (TBAB) was supplied by Sigma Aldrich (St. Louis, MO, USA) and used as a catalyst for the cycloaddition of carbon dioxide (CO_2_) and transfer agent for the epoxidation reaction. In addition, 1,3-diaminopropane was purchased from Merck, and 1,8-Diazabicyclo[5.4.0]undec-7-ene (DBU) and hexagonal boron nitride (h-BN) (particle size~1 µm) were acquired from Sigma Aldrich. H-Boron nitride was functionalized with commercial sugar to enhance mechanical properties.

### 2.2. Synthesis

#### 2.2.1. Preparation of Bi-Cyclic Carbonate

Typically, a commercial epoxide (70 g) was added to a 250-mL glass flask by using TBAB (3.5 g) as a catalyst, and CO_2_ gas is bubbled into the glass flask under atmospheric pressure and equilibrated at 105 °C, without the use of an autoclave. Vacuum distillation was used to separate the pure cyclic carbonates when the reaction was completed. The conversion of epoxides to cyclic carbonate was followed kinetically through Fourier transform infrared (FTIR) analysis to optimize the reaction conditions and therefore to obtain a high conversion rate in a short reaction time.

#### 2.2.2. Functionalization of Hexagonal Boron Nitride

Hexagonal boron nitride sheets were simultaneously exfoliated and functionalized by a typical sugar-assisted mechanochemical exfoliation (SAMCE) process [29]. Raw h-BN (2 g), sucrose crystals (10 g), and steel balls with diameters of 10 mm were cryo-grinded for 1 h (number of cycles = 20, grinding time = 2 min, intermediate cooling time = 1 min) in a cryomill (Retsch, Hann, Germany). The milled mixture was washed with 200 mL deionized water and filtered through a Teflon membrane (0.2 μm pore size) under vacuum. This process was repeated four times to thoroughly remove free sucrose and obtained the f-BN.

#### 2.2.3. Preparation of PHU Films and Nanocomposites

PHU films were produced from the reaction of the bi-cyclic carbonate, diaminopropane and DBU. The bi-cyclic carbonate (5.0 g) was added into a beaker followed by the diaminopropane (2.5 g) and the DBU (0.25 g) and blended manually. They were then vacuum-degassed for 5 min and poured in a Teflon mold. Finally, the mixture was introduced in an oven at 95 °C for 12 h. PHU/f-BN films were prepared by first dispersing the nanofiller in THF (5 mg/mL), which was added to the bi-cyclic carbonate and diaminopropane mixture prior to the inclusion of the DBU.

### 2.3. Methods

The kinetics of bi-cyclic carbonate formation were monitored by Fourier transform infrared spectroscopy (FTIR). The FTIR spectra were recorded by using a PerkinElmer Spectrum One. An FTIR spectrometer was fitted with an attenuated total reflectance (ATR) accessory under unforced conditions. The reactive mixture was placed in direct contact with the diamond crystal each hour. Infrared spectra for PHU films were collected in the range from 450 to 4000 cm^−1^ with a resolution of 4 cm^−1^ and four scans per spectrum were co-added.

Raman spectroscopy was performed on a Renishaw 2000 Confocal Raman Microprobe by using a 514.5 nm argon ion laser and 0.02 cm^−1^ resolution. The spectra were recorded from 750 to 3500 cm^−1^.

Thermogravimetric analysis was carried out by using a TA-Q500. Samples of 10 mg were placed on platinum dishes and heated under a nitrogen atmosphere (flow rate 90 mL/min) from room temperature to 800 °C at 10 °C/min. The results were analyzed in TA Instruments’ Universal Analysis software.

Dynamic mechanical analysis was performed on a DMA Q800 from TA Instruments. Temperature sweeps from −100 °C to 70 °C, heating rate of 2 °C/min were performed in tension mode with an amplitude of 15 µm and a frequency of 1 Hz. The glass transition was recorded from the maximum of the damping factor, tan δ.

Glass transition temperature (T_g_) was measured on a differential scanning calorimetry (NETZSH DSC- 214 model), in the temperature range from −50 to 120 °C, and at heating rate of 10 °C/min under nitrogen atmosphere. The stability of baseline was checked before each measurement.

Scanning electron microscopy was performed on a Philips model XL30 with tungsten filament and accelerating voltage of 25 kV, and was used to examine the morphology of the films. Samples were cryo-fractured under liquid nitrogen and coated with Au-Pd in a sputter coated Polaron SC7640 prior to observation.

## 3. Results and Discussion

### 3.1. Synthesis of Bi-Cyclic Five-Membered Carbonate

**Scheme 1:**
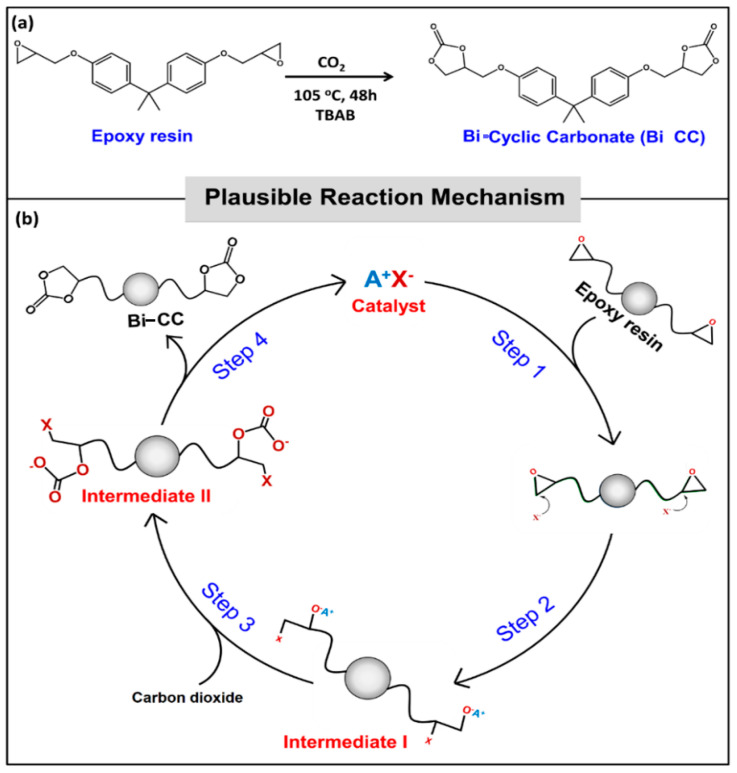
(**a**) Synthesis of bi-cyclic carbonate with CO_2_ at atmospheric pressure. (**b**) Plausible reaction mechanism of the insertion of CO_2_.

**Figure 1:**
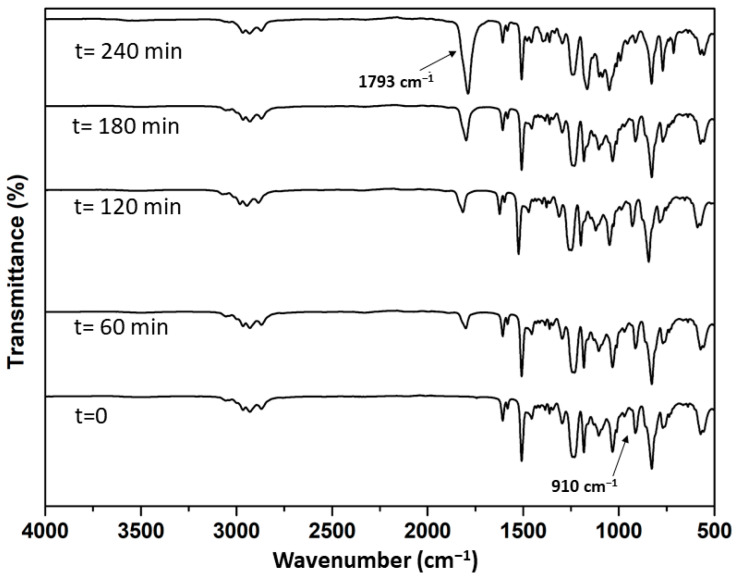
ATR-FTIR spectra of different time for the synthesis of cyclic carbonate from commercial epoxy.

The bi-cyclic carbonate was prepared by a reaction of commercially available epoxide and CO_2_. Typically, the reaction was carried out in glass flask by using TBAB as a catalyst and CO_2_ gas under atmospheric pressure. The mechanism for this reaction has already been reported and is described by the ring opening of the epoxide by the bromide ion [30]. The addition of CO_2_ into oxiranes at high temperatures and pressures is the most commonly used technique for synthesis of cyclic carbonate. Here, the bi-cyclic carbonate was prepared at atmospheric pressure without the use of an autoclave (Scheme 1). Typically, the reaction was carried out by using commercial bisphenol A-based epoxy resin and CO_2_ in a glass flask in the presence of TBAB as a catalyst. The progress of the reaction was monitored and optimized by FTIR (Figure 1).

FTIR analysis reveals that the conversion is almost complete after a reaction time of 4 h (Figure 1), from the progressive disappearance of the characteristic epoxide band at 910 cm^−1^ and the increase of the carbonyl band of the formed cyclic carbonate at 1793 cm^−1^. Under normal pressure, the yield of the coupling of CO_2_ with epoxides to cyclic carbonates after 4 h of reaction reached 82.3% by using TBAB as a catalyst. Further synthesis experiments were carried out for a longer reaction time, 24 h, and led to a yield not exceeding 83%. This result is comparable to previously reported works [31,32,33] and indicates the high activity of TBAB compared to various heterogeneous catalysts, such as Mg-Al mixed oxides [34], smectites [35], and iron-based composites [36] that have shown yields between 41% and 75%. 

### 3.2. Functionalization of Boron Nitride

**Figure 2:**
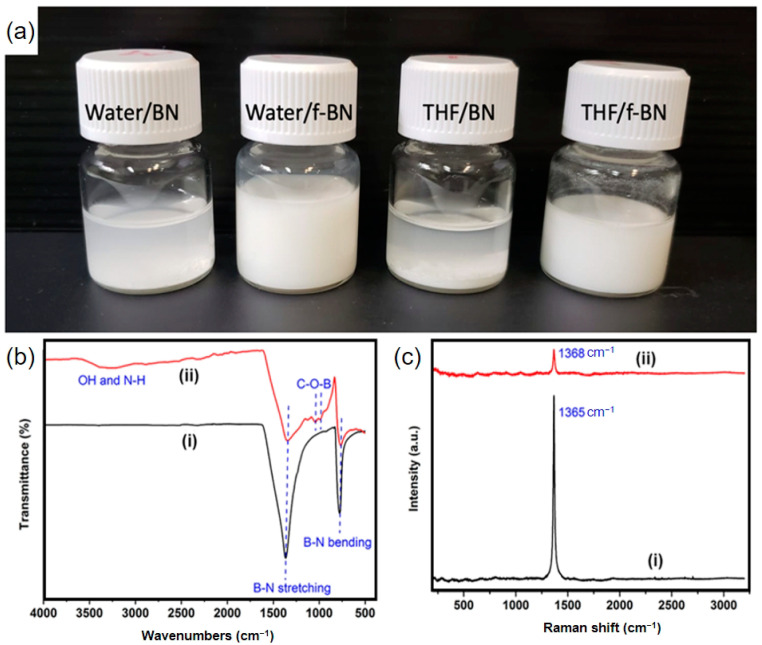
(**a**) Dispersion of h-BN and f-BN in water and THF. (**b**) ATR-FTIR and (**c**) Raman of the (i) h-BN and (ii) functionalized BN.

The sugar-assisted mechanochemical exfoliation (SAMCE) technique [29] leads to the simultaneous exfoliation and functionalization of hexagonal boron nitride sheets. Functionalized BN (f-BN) particles present a good and stable dispersion in water and THF at concentrations up to 25.0 mg g^−1^ (Figure 2a). FTIR spectra of the h-BN and f-BN (Figure 2b) show the presence of B-N bending (≈750 cm^−1^) and stretching (≈1320 cm^−1^) peaks and of additional peaks of hydroxyl, amine, and multiple peaks in the region of 1300–800 cm^−1^ for sugar molecules. Raman spectra of the h-BN and f-BN also suggested that the crystal and phase structures are retained after mechanochemical exfoliation (Figure 2c). Similar results were reported by Chen et al. [29] that suggested that ball milling in the presence of sucrose crystals cleaved the h-BN platelets into small thin flakes with active N and B edges and stabilized surfaces, all covered by sucrose molecules. 

### 3.3. PHU Nanocomposite Films

After the synthesis of bi-cyclic carbonate by using epoxy resin and CO_2_ and the functionalization of hexagonal boron nitride, PHU thermoplastic films were prepared by using three different weight percentages of functionalized BN (1, 2, and 3%). The polymerization is done without solvent to obtain better properties of the films and to decrease of monomer concentration leading to reduction of reaction rate to reduce the impact of solvents on the environment. The obtained nanocomposites films were studied by using different characterization methods: ATR-FTIR, TGA, DMA, and DSC.

**Figure 3:**
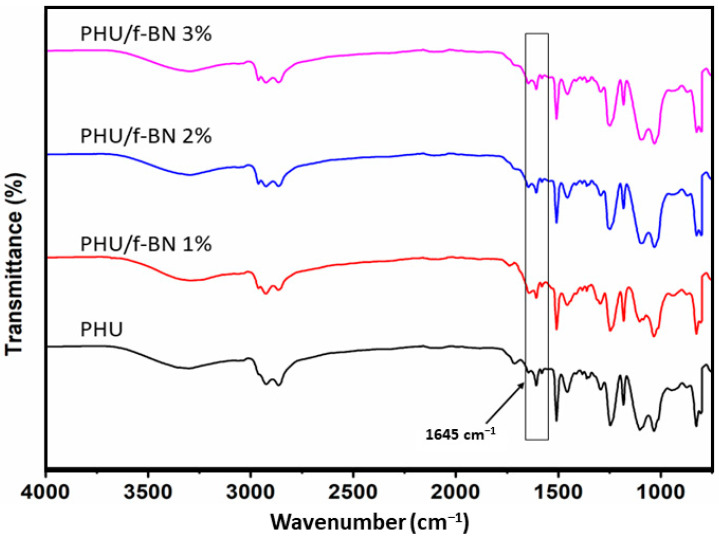
ATR-FTIR of different PHU films.

The ATR-FTIR analysis was performed to check the conversion of carbonate groups of bi-cyclic carbonate to PHU. Figure 3 shows the overlaid infrared spectra of all films made with and without various percentages of functionalized boron nitride. All the films have comparable spectra. Indeed, in the spectra of PHU and PHU/f-BN we observe the total absence of the peaks related to the carbonyl group of the cyclic carbonate, at 1793 cm^−1^, indicating its complete conversion into urethane which is evidenced by the presence of the stretching of the C=O group of the urethane band at 1645 cm^−1^. Between 3200 and 3600 cm^−1^, a broad band is observed, indicating the presence of NH groups of the urethane functions and the hydroxyl groups resulting from the opening of the cyclic carbonate by the amine functions.

**Figure 4:**
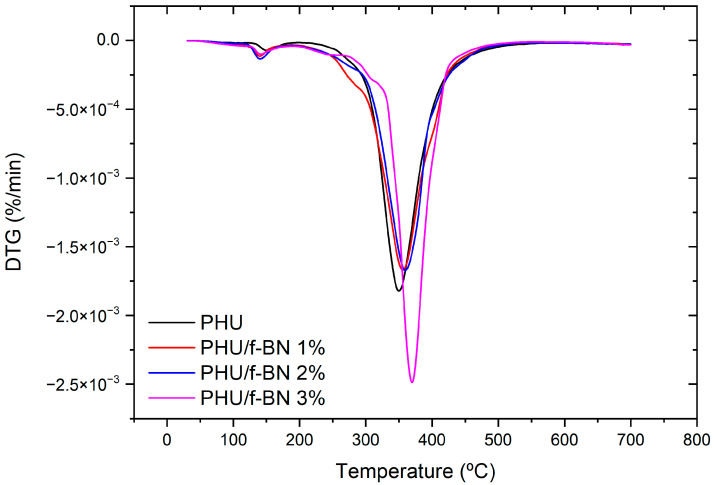
Thermogravimetric analysis (TGA) curves of different thermoplastic PHU films.

Thermogravimetric analysis was used to determine the thermal stability of the various nanocomposite films of PHUs compared to neat PHU. As shown by Figure 4, anunfilled PHU sample degrades within the range 250–450 °C, with the maximum degradation rate occurring at 350 °C (Table 1). The degradation presents an initial weight loss between 100 °C and 150 °C prior to the major degradation step, which could be ascribed to the evaporation of absorbed moisture or of residual monomers [37,38]. Thermal degradation of PHUs has been reported over a wide temperature range from as low as 180 °C up to 388 °C depending on the size of the aromatic part. Thus, the synthesized PHU presents good thermal stability, which lies well within the values of conventional PU [39]. The main decomposition of PHU has been ascribed to the dissociation of the urethane bond [38]. The addition of f-BN does not modify the degradation kinetics [27] but improves the thermal stability of PHU at approximately 20 °C, increasing the temperature at the maximum degradation rate to 370 °C for the 3 wt.% f-BN (Table 1). This effect is widely reported in the literature as is ascribed to diffusion and transmission rate of oxygen [27,40].

**Figure 5:**
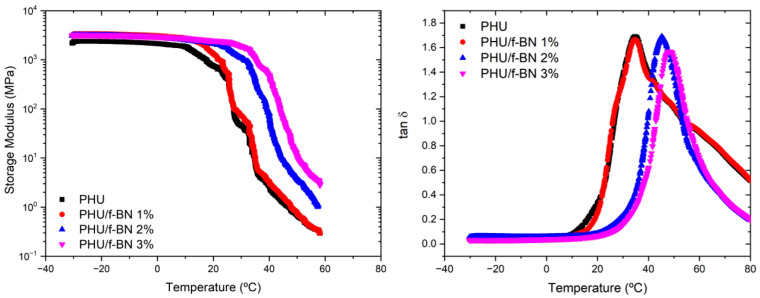
Storage modulus and damping factor (tan δ) of the PHU films.

The thermomechanical characteristics of nanocomposites PHU films were studied by using DMA. Figure 5 shows the storage modulus (E’) and loss factor (tan δ) as a function of temperature for all PHU films. At temperatures above T_g_, E’ increases with f-BN content from 7.8 MPa for the PHU film up to 11.6 MPa for the PHU/f-BN 3% (Table 1). The presence of f-BN restricts the mobility of the rubber chains, increasing the stiffness of the polymer [24,41,42]. It is well known that the E’ of a filled polymer composite is influenced by the effective interfacial interaction between the polymer matrix and the filler particles. In general, a strong interfacial interaction results in a high E’ [43]. Meanwhile, the tan δ (Figure 5) shows a shift of the T_g_ from 31.7 °C up to 49.3 °C with increasing f-BN content (Table 1) indicative of a reduction of the molecular chain mobility. Furthermore, a broad glass-to-rubber transition is commonly attributed to the presence of a large distribution in the molecular weight between cross-links or some other type of heterogeneity in the network structure [44]. Thus, the presence of f-BN appears to increase not only the cross-link density but also the homogeneity of the network structure. Similar shifts in T_g_ have been reported in PHU filled with CNTs [20], polyhedral oligomeric silsesquioxanes [16], and graphenated ceramic fillers [24].

**Figure 6:**
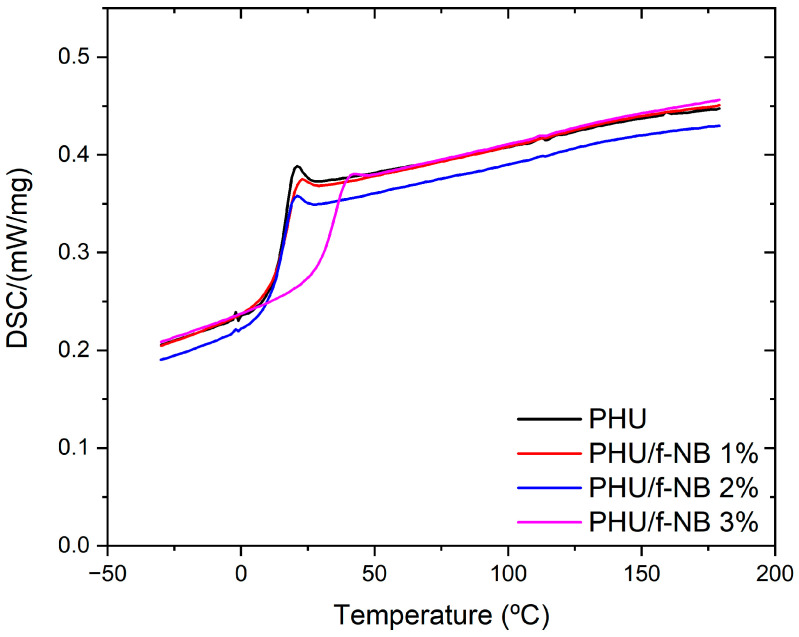
Differential scanning calorimetry (DSC) curves of all PHUs films.

The DSC curves of PHU films show comparable patterns with no melting of a crystalline phase during the second heating, suggesting that all systems are amorphous (Figure 6). The films with different percentages of f-BN present moderate to strong shifts in the glass transition temperatures (Table 1), corroborating the DMA results. As discussed previously, the shift is ascribed to both the presence of the f-BN restricting the segmental motion and cross-linking of the molecular network [18] ascribed to intramolecular hydrogen bonding between the functional groups, i.e., hydroxyl, ethers, carbonyl, and the urethane bonds [45].

**Figure 7:**
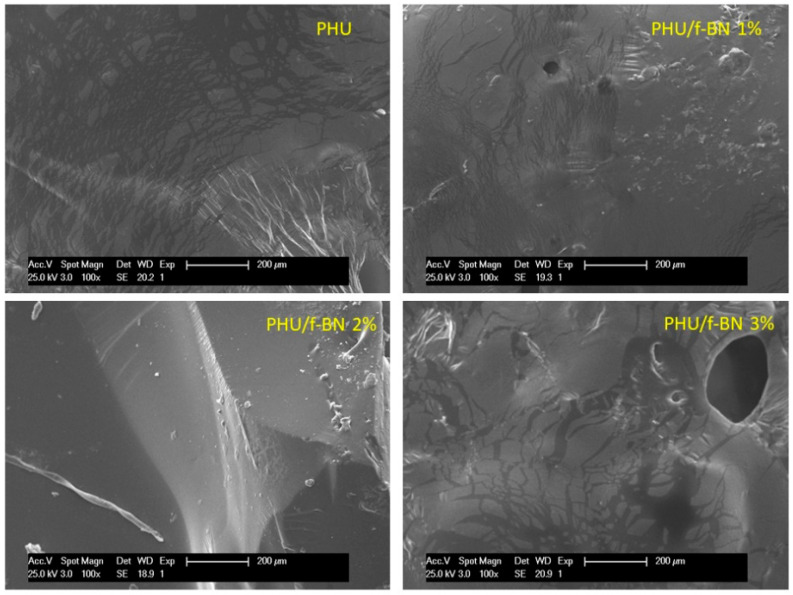
SEM images of PHU polymer and PHU/f-BN composites.

SEM images of PHU and PHU/f-BN composites are shown in Figure 7. Micrographs of the composites show no phase separation and agglomeration of f-BN, indicating the nanoparticles are well dispersed in the polymer matrix.

## 4. Conclusions

In this study, we have explored the synthesis of cyclic carbonate monomer and its polymerization to polyhydroxyurethane (PHU). Furthermore, we analyzed the use of functionalized boron nitrate as a strategy to improve the thermal and mechanical properties of the matrix (PHU). The optimized synthesis route is an industrially scalable process, under atmospheric pressure and mild conditions with a high yield, which results in PHU with properties comparable to conventional PU. The use of a solvent-free, mechanochemical process provides a clean methodology by which to functionalize and exfoliate hexagonal boron nitrate with hydroxyl and amine groups, improving its interaction with the polymer chains. Additionally, the inclusion of f-BN appears to reduce the heterogeneity of the network, improving the thermal and mechanical properties in terms of the thermal degradation temperature, glass transition temperature, and the modulus. Therefore, the developed PHU composites are an efficient, environmentally friendly, and industrially scalable material that can be a suitable alternative to polyurethanes.

## Figures and Tables

**Table 1:** Different parameters of all PHU films.

	T_d_ (°C) *	T_g_ (°C) **	T_α_ (°C) ***	E’ (MPa) ***
PHU	350	18.5	31.7	7.8
PHU/f-BN 1%	356	19.1	31.8	8.1
PHU/f-BN 2%	360	19.8	42.5	9.8
PHU/f-BN 3%	370	29.2	49.3	11.6

T_d_: Temperature at maximum degradation rate, E’: storage modulus at T in rubbery region, * Measured by TGA, ** Measured by DSC, *** Measured by DMA.

## Data Availability

The data that support the findings of this study are available from the corresponding author, RV.

## Abbreviations

Poly(hydroxyurethane) (PHU), Polyurethane (PU), Hexagonal Boron Nitrate (h-BN), Functionalized Boron Nitrate (f-BN), Non-isocyanate polyurethane (NIPU), Carbon dioxide (CO_2_), sugar-assisted mechanochemical exfoliation (SAMCE), Tetrabutylammonium bromide (TBAB), 1,8-Diazabicyclo[5.4.0]undec-7-ene (DBU), Tetrahydrofuran (THF), Thermogravimetric analysis (TGA), Dynamic mechanical analysis (DMA), Differential scanning calorimetry (DSC), Fourier transform infrared spectroscopy (FTIR), Attenuated total reflectance (ATR), Glass transition temperature (T_g_), Storage modulus (E’), Loss factor (tan δ), Scanning electron microscopy (SEM).

## References

[1] Akindoyo J.O., Beg M.D.H., Ghazali S., Islam M.R., Jeyaratnam N., Yuvaraj A.R. (2016). Polyurethane Types, Synthesis and Ap-plications—A Review. RSC Adv..

[2] Blattmann H., Fleischer M., Bähr M., Mülhaupt R. (2014). Isocyanate- and Phosgene-Free Routes to Polyfunctional Cyclic Carbonates and Green Polyurethanes by Fixation of Carbon Dioxide. Macromol. Rapid Commun..

[3] Gomez-Lopez A., Elizalde F., Calvo I., Sardon H. (2021). Trends in Non-Isocyanate Polyurethane (NIPU) Development. Chem. Commun..

[4] Maisonneuve L., Lamarzelle O., Rix E., Grau E., Cramail H. (2015). Isocyanate-Free Routes to Polyurethanes and Poly(Hydroxy Urethane)S. Chem. Rev..

[5] Kathalewar M.S., Joshi P.B., Sabnis A.S., Malshe V.C. (2013). Non-Isocyanate Polyurethanes: From Chemistry to Applications. RSC Adv..

[6] Martínez de Sarasa Buchaca M., de la Cruz-Martínez F., Francés-Poveda E., Fernández-Baeza J., Sánchez-Barba L.F., Garcés A., Castro-Osma J.A., Lara-Sánchez A. (2022). Synthesis of Nonisocyanate Poly(hydroxy)urethanes from Bis(cyclic carbonates) and Polyamines. Polymers.

[7] Alves M., Grignard B., Mereau R., Jerome C., Tassaing T., Detrembleur C. (2017). Organocatalyzed Coupling of Carbon Dioxide with Epoxides for the Synthesis of Cyclic Carbonates: Catalyst Design and Mechanistic Studies. Catal. Sci. Technol..

[8] Claver C., Yeamin M.B., Reguero M., Masdeu-Bultó A.M. (2020). Recent Advances in the Use of Catalysts Based on Natural Products for the Conversion of CO_2_ into Cyclic Carbonates. Green Chem..

[9] Song B., Qin A., Tang B.Z. (2022). Syntheses, Properties, and Applications of CO_2_-Based Functional Polymers. Cell Rep. Phys. Sci..

[10] Stachak P., Łukaszewska I., Hebda E., Pielichowski K. (2021). Recent Advances in Fabrication of non-isocyanate Polyure-thane-based composite materials. Materials.

[11] Ecochard Y., Caillol S. (2020). Hybrid polyhydroxyurethanes: How to overcome limitations and reach cutting edge properties?. Eur. Polym. J..

[12] Panchireddy S., Grignard B., Thomassin J.M., Jerome C., Detrembleur C. (2018). Bio-Based Poly(Hydroxyurethane) Glues for Metal Substrates. Polym. Chem..

[13] Panchireddy S., Thomassin J.M., Grignard B., Damblon C., Tatton A., Jerome C., Detrembleur C. (2017). Reinforced Poly(Hydroxyurethane) Thermosets as High Performance Adhesives for Aluminum Substrates. Polym. Chem..

[14] Türünç O., Kayaman-Apohan N., Kahraman M.V., Menceloǧlu Y., Güngör A. (2008). Nonisocyanate Based Polyurethane/Silica Nanocomposites and Their Coating Performance. J. Sol-Gel Sci. Technol..

[15] Blattmann H., Mülhaupt R. (2016). Multifunctional POSS Cyclic Carbonates and Non-Isocyanate Polyhydroxyurethane Hybrid Materials. Macromol..

[16] Liu W., Hang G., Mei H., Li L., Zheng S. (2022). Nanocomposites of Polyhydroxyurethane with POSS Microdomains: Synthesis via Non-Isocyanate Approach, Morphologies and Reprocessing Properties. Polymers.

[17] Kathalewar M., Sabnis A., Waghoo G. (2013). Effect of incorporation of surface treated zinc oxide on non-isocyanate polyurethane based nano-composite coatings. Prog. Org. Coat..

[18] Li L., Zhao B., Wang H., Gao Y., Hu J., Zheng S. (2021). Nanocomposites of Polyhydroxyurethane with Fe3O4 Nanoparticles: Synthesis, Shape Memory and Reprocessing Properties. Compos. Sci. Technol..

[19] He X., Xu X., Bo G., Yan Y. (2020). Studies on the Effects of Different Multiwalled Carbon Nanotube Functionalization Techniques on the Properties of Bio-Based Hybrid Non-Isocyanate Polyurethane. RSC Adv..

[20] Adeel M., Zhao B., Li L., Zheng S. (2020). Nanocomposites of Poly(Hydroxyurethane)s with Multiwalled Carbon Nanotubes: Synthesis, Shape Memory, and Reprocessing Properties. ACS Appl. Polym. Mater..

[21] Ge W., Zhao B., Li L., Nie K., Zheng S. (2021). Nanocomposites of Polyhydroxyurethane with Nanocrystalline Cellulose: Syn-thesis, Thermomechanical and Reprocessing Properties. Eur. Polym. J..

[22] Białkowsk A., Bakar M., Przybyłek M. (2018). Effect of Nonisocyanate Polyurethane and Nanoclay on the Mechanical Properties of an Epoxy Resin. Mech. Compos. Mater..

[23] Gennen S., Grignard B., Thomassin J.M., Gilbert B., Vertruyen B., Jerome C., Detrembleur C. (2016). Polyhydroxyurethane Hydrogels: Synthesis and Characterizations. Eur. Polym. J..

[24] Yang Y., Pössel B., Mülhaupt R. (2020). Graphenated Ceramic Particles as Functional Fillers for Nonisocyanate Polyhydroxyure-thane Composites. Macromol. Mater. Eng..

[25] Doley S., Sarmah A., Sarkar C., Dolui S.K. (2018). In Situ Development of Bio-Based Polyurethane-Blend-Epoxy Hybrid Materials and Their Nanocomposites with Modified Graphene Oxide via Non-Isocyanate Route. Polym. Int..

[26] Meziani M.J., Sheriff K., Parajuli P., Priego P., Bhattacharya S., Rao A.M., Quimby J.L., Qiao R., Wang P., Hwu S.-J. (2022). Advances in Studies of Boron Nitride Nanosheets and Nanocomposites for Thermal Transport and Related Applications. ChemPhysChem.

[27] Rasul M.G., Kiziltas A., Arfaei B., Shahbazian-Yassar R. (2021). 2D Boron Nitride Nanosheets for Polymer Composite Materials. npj 2D Mater. Appl..

[28] Hayat A., Sohail M., Hamdy M.S., Taha T.A., AlSalem H.S., Alenad A.M., Amin M.A., Shah R., Palamanit A., Khan J. (2022). Fabrication, Characteristics, and Applications of Boron Nitride and Their Composite Nanomaterials. Surf. Interfaces.

[29] Chen S., Xu R., Liu J., Zou X., Qiu L., Kang F., Liu B., Cheng H.M. (2019). Simultaneous Production and Functionalization of Boron Nitride Nanosheets by Sugar-Assisted Mechanochemical Exfoliation. Adv. Mater..

[30] Caló V., Nacci A., Monopoli A., Fanizzi A. (2002). Cyclic Carbonate Formation from Carbon Dioxide and Oxiranes in Tetrabu-tylammonium Halides as Solvents and Catalysts. Org. Lett..

[31] North M., Pasquale R. (2009). Mechanism of Cyclic Carbonate Synthesis from Epoxides and CO2. Angew. Chemie-Int. Ed..

[32] Castro-Osma J.A., North M., Wu X. (2016). Synthesis of Cyclic Carbonates Catalysed by Chromium and Aluminium Salphen Complexes. Chem.-Eur. J..

[33] Ohkawara T., Suzuki K., Nakano K., Mori S., Nozaki K. (2014). Facile Estimation of Catalytic Activity and Selectivities in Co-polymerization of Propylene Oxide with Carbon Dioxide Mediated by Metal Complexes with Planar Tetradentate Ligand. J. Am. Chem. Soc..

[34] Yamaguchi K., Ebitani K., Yoshida T., Yoshida H., Kaneda K. (1999). Mg-Al Mixed Oxides as Highly Active Acid-Base Catalysts for Cycloaddition of Carbon Dioxide to Epoxides. J. Am. Chem. Soc..

[35] Dai W.L., Luo S.L., Yin S.F., Au C.T. (2009). The Direct Transformation of Carbon Dioxide to Organic Carbonates over Heteroge-neous Catalysts. Appl. Catal. A Gen..

[36] Qu J., Cao C.Y., Dou Z.F., Liu H., Yu Y., Li P., Song W.G. (2012). Synthesis of Cyclic Carbonates: Catalysis by an Iron-Based Composite and the Role of Hydrogen Bonding at the Solid/Liquid Interface. ChemSusChem.

[37] Carré C., Bonnet L., Avérous L. (2014). Original Biobased Nonisocyanate Polyurethanes: Solvent- and Catalyst-Free Synthesis, Thermal Properties and Rheological Behaviour. RSC Adv..

[38] Van Velthoven J.L.J., Gootjes L., Van Es D.S., Noordover B.A.J., Meuldijk J. (2015). Poly(Hydroxy Urethane)s Based on Renewable Diglycerol Dicarbonate. Eur. Polym. J..

[39] Bernal M.M., Molenberg I., Estravis S., Rodriguez-Perez M.A., Huynen I., Lopez-Manchado M.A., Verdejo R. (2012). Comparing the Effect of Carbon-Based Nanofillers on the Physical Properties of Flexible Polyurethane Foams. J. Mater. Sci..

[40] Yin S., Ren X., Lian P., Zhu Y., Mei Y. (2020). Synergistic Effects of Black Phosphorus/Boron Nitride Nanosheets on Enhancing the Flame-Retardant Properties of Waterborne Polyurethane and Its Flame-Retardant Mechanism. Polymers.

[41] Kim K., Kim M., Kim J. (2014). Enhancement of the Thermal and Mechanical Properties of a Surface-Modi Fi Ed Boron Nitride–Polyurethane Composite. Polym. Adv. Technol..

[42] Kim K., Kim M., Kim J. (2014). Fabrication of UV-Curable Polyurethane Acrylate Composites Containing Surface-Modified Boron Nitride for Underwater Sonar Encapsulant Application. Ceram. Int..

[43] Bashir M.A. (2021). Use of Dynamic Mechanical Analysis (DMA) for Characterizing Interfacial Interactions in Filled Polymers. Solids.

[44] Landel R.F., Nielsen L.E. (1994). Mechanical Properties of Polymers and Composites.

[45] Gholami M., Shakeri A., Zolghadr M., Yamini G. (2021). Non-Isocyanate Polyurethane from the Extracted Tannin of Sumac Leaves: Synthesis, Characterization, and Optimization of the Reaction Parameters. Ind. Crops Prod..

